# *Cryptococcus neoformans* trehalose-6-phosphate synthase (tps1) promotes organ-specific virulence and fungal protection against multiple lines of host defenses

**DOI:** 10.3389/fcimb.2024.1392015

**Published:** 2024-05-22

**Authors:** Kristie Goughenour, Arianna Creech, Jintao Xu, Xiumiao He, Rylan Hissong, Charles Giamberardino, Jennifer Tenor, Dena Toffaletti, John Perfect, Michal Olszewski

**Affiliations:** ^1^ Research Service, Lieutenant Colonel Charles S. Kettles VA Medical Center, Ann Arbor, MI, United States; ^2^ Division of Pulmonary and Critical Care Medicine, Department of Internal Medicine, University of Michigan, Ann Arbor, MI, United States; ^3^ Division of Infectious Diseases, Department of Medicine, Duke University, Durham, NC, United States

**Keywords:** *Cryptococcus*, trehalose, TPS1, capsule, pulmonary, disseminated

## Abstract

Trehalose-6-phosphate synthase (TPS1) was identified as a virulence factor for *Cryptococcus neoformans* and a promising therapeutic target. This study reveals previously unknown roles of TPS1 in evasion of host defenses during pulmonary and disseminated phases of infection. In the pulmonary infection model, TPS1-deleted (*tps1Δ*) *Cryptococci* are rapidly cleared by mouse lungs whereas TPS1-sufficent WT (H99) and revertant (*tps1Δ*:*TPS1*) strains expand in the lungs and disseminate, causing 100% mortality. Rapid pulmonary clearance of *tps1Δ* mutant is T-cell independent and relies on its susceptibility to lung resident factors and innate immune factors, exemplified by *tps1Δ* but not H99 inhibition in a coculture with dispersed lung cells and its rapid clearance coinciding with innate leukocyte infiltration. In the disseminated model of infection, which bypasses initial lung–fungus interactions, *tps1Δ* strain remains highly attenuated. Specifically, *tps1Δ* mutant is unable to colonize the lungs from the bloodstream or expand in spleens but is capable of crossing into the brain, where it remains controlled even in the absence of T cells. In contrast, strains H99 and *tps1Δ:TPS1* rapidly expand in all studied organs, leading to rapid death of the infected mice. Since the rapid pulmonary clearance of *tps1Δ* mutant resembles a response to acapsular strains, the effect of *tps1* deletion on capsule formation *in vitro* and *in vivo* was examined. *Tps1Δ* cryptococci form capsules but with a substantially reduced size. In conclusion, TPS1 is an important virulence factor, allowing *C. neoformans* evasion of resident pulmonary and innate defense mechanisms, most likely *via* its role in cryptococcal capsule formation.

## Introduction

*Cryptococcus neoformans* is an ubiquitous environmental fungus and a major human fungal pathogen, causing an estimated 180,000 annual deaths (Rajasingham et al., 2017). *Cryptococcus* generally acts as an opportunistic pathogen, infecting those with immune deficiencies, especially defects in T-cell responses associated with HIV infections, immune-suppressive therapies, or other underlying causes (Baddley et al., 2008; Rajasingham et al., 2017). *C. neoformans* enters the host *via* the respiratory tract, and the ability of the fungi to survive in the lung environment is critical to the pathogen’s success (May et al., 2016; Ballou and Johnston, 2017). Breaching pulmonary defenses enables *C. neoformans* to disseminate to the brain and result in the highly lethal cryptococcal meningoencephalitis (Tugume et al., 2023). In contrast, the generation of a protective T-cell-mediated response can successfully contain and eliminate the fungus in the lungs and prevent dissemination (Lim and Murphy, 1980; Mody et al., 1990, 1993; Huffnagle et al., 1991; Garcia-Hermoso et al., 1999; Goldman et al., 2000). Thus, the way the immune system responds to *C. neoformans* modulates the balance between fungal control and fungal spread to and colonization of multiple organs.

Apart from the underlying immune deficiencies and high propensity of the fungus to infect the brain, the high mortality rate in cryptococcosis patients is linked to limited therapeutic options to a handful of drug classes (Perfect et al., 2010; Roemer and Krysan, 2014; Krysan, 2017; McKeny et al., 2022). These antifungals have well-established toxicity problems (McKeny et al., 2022), limiting their clinical use or at least requiring careful monitoring. The newest antifungals, the echinocandins, which have significantly improved toxicity profiles, unfortunately are not effective against *Cryptococcus* (Wiederhold et al., 2013; Roemer and Krysan, 2014). As such, current treatments of *Cryptococcus* rely on long treatments using older problematic antifungals (Perfect et al., 2010) and cryptococcal resistance to antifungals has been observed, leading to measurable relapses (Cheong and McCormack, 2013; Bongomin et al., 2017). These challenges have been highlighted by the World Health Organization, which placed *C. neoformans* in their critical priority group for pathogenic fungi (WHO fungal priority pathogens list to guide research, development and public health action, 2022), which summarizes and supports the critical need for the development of new therapeutic approaches, targeting fungal pathways that are essential for fungal virulence and/or host defense evasion that enables fungal persistence.

The trehalose biosynthetic pathway has received attention as a potential antifungal target (Perfect et al., 2016). Trehalose is a disaccharide produced by *C. neoformans* and *C. gattii* that is known to protect against abiotic stress (Richards et al., 2002; Furuki et al., 2009). Trehalose is biosynthesized *via* a two-step process with trehalose-6-phosphate synthase (TPS1) catalyzing the first step and producing a major regulatory product, trehalose-6-phosphates. Loss of this first step is sufficient to prevent the production of trehalose (Petzold et al., 2006). Deletion of *tps1* in *C. neoformans* results in loss of virulence in murine, immunosuppressed rabbit, and *C. elegans* models (Petzold et al., 2006) and *C. gattii* in mice (Ngamskulrungroj et al., 2009). The *C. gattii* trehalose-deficient strain (*tps1Δ*) shows a defective response to heat stress, oxidation, and dehydration (Petzold et al., 2006). However, it is not understood where and how TPS1 is required only for virulence in these models. Previous work has established that the avirulence seen is not due to thermosensitivity (Petzold et al., 2006). Therefore, the question remains of how the loss of TPS1 renders *C. neoformans* and *C. gattii* unable to cause a lethal infection. It is unknown if TPS1 is required only for fungal survival in the host or for the establishment of subclinical or latent infections. Even less is understood about how *tps1* deletion alters interactions of the fungi with the host. What host defenses that could contribute to the control of *tps1*-deleted *C. neoformans* is not known.

In this study, we found that interception of the cryptococcal trehalose pathway achieved *via tps1*-gene deletion in *tps1Δ* strain waved the requirement for CD4+ T cells for *C. neoformans* clearance. We also found a crucial role for TPS1 to withstand pulmonary fungal defenses including the resident mechanisms already present in the uninfected lungs. Finally, we linked these findings with the role of TPS1 in *C. neoformans* capsule formation, which likely contributed to the avirulent phenotype of *tps1Δ*.

## Materials and methods

### Mice

BALB/c (stock # 000651) and C57BL/6J (stock #:000664) background mice were purchased from Jackson Laboratory (Bar Harbor, ME) and housed in a specific pathogen-free environment at the Ann Arbor Veterans Affairs Medical Center. Male and female mice were infected at 8–15 weeks of age and were humanely euthanized *via* CO_2_ inhalation. All experiments in this study were performed per National Institutes of Health guidelines and the Guide for the Care and Use of Laboratory Animals and approved by the Veterans Affairs Institutional Animal Care and Use Committee (protocol 1702709, 1597346). For mortality experiments, mice were euthanized at 80% of their body weight after infection. Mice were also monitored for neurological symptoms although none were observed.

### *C. neoformans* infection

**Figure 1:**
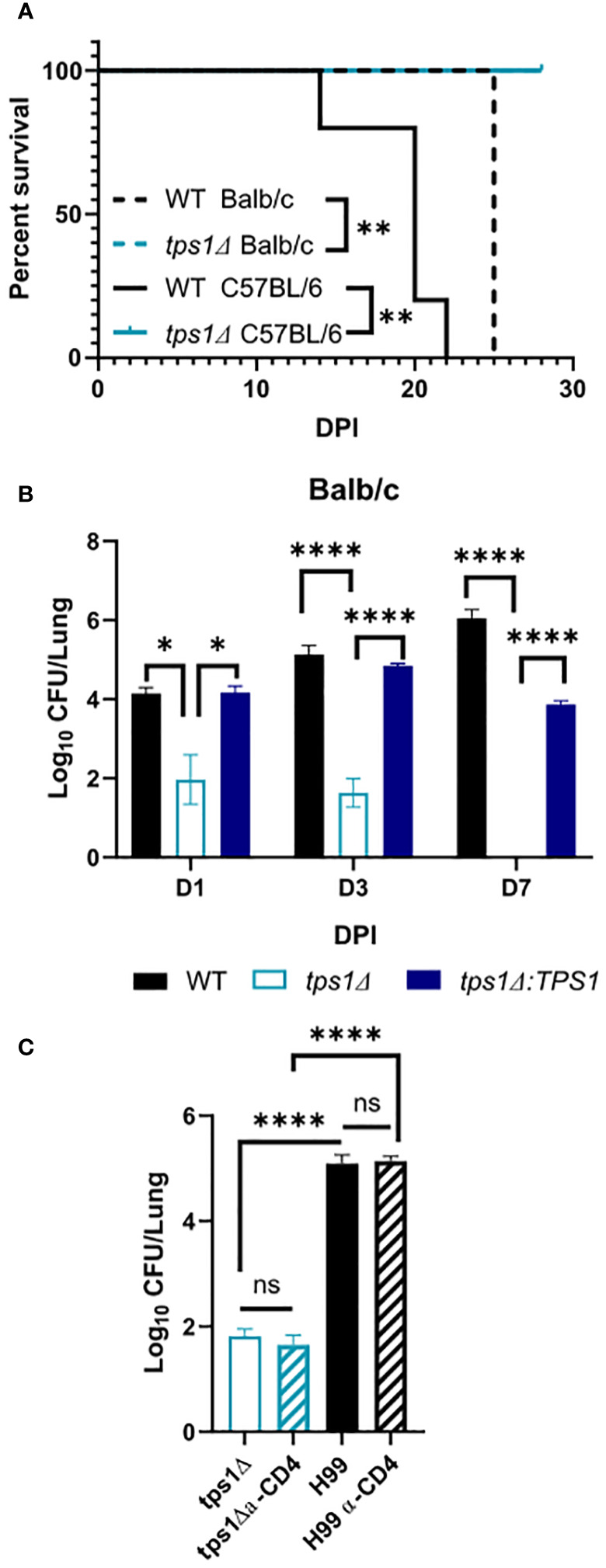
Cryptococcal trehalose phosphate synthase 1 (*tps1*) gene deletion enables rapid reduction of pulmonary fungal burden *via* a T-cell-independent mechanism. C57BL/6 and BALB/c mice were infected intratracheally (IT) with 1*10^5^ WT (H99), tps1 KO (*tps1Δ*), or complemented (*tps1Δ*:*TPS1*) *C. neoformans* (*Cn*). **(A)** The *Cn-*infected mice were monitored for 28 days postinfection (dpi) for survival. All the H99-infected mice reached endpoint criteria between 14 dpi and 25 dpi, whereas all *tps1Δ*-infected mice survived. Data from a matched experiment; n = 5 mice/group. **(B)** Pulmonary fungal burdens were evaluated at 1 dpi, 3 dpi, and 7 dpi. Data are pooled from at least three matched experiments (n = 2–6 mice/group). **(C)** Lung fungal burdens at 3 dpi in *tps1Δ*-infected BALB/c mice treated with anti-CD4 depleting antibody or control (PBS) injections. Data from two pooled experiments; n = 2–5 mice/group. Data in all figures are the means ± SEM (unless otherwise indicated) and analyzed as described in the methods section. Statistically significant differences are indicated in all figures as follows: *p ≤ 0.05, **p ≤ 0.01, ****p ≤ 0.0001. NS, no significance.

**Figure 2:**
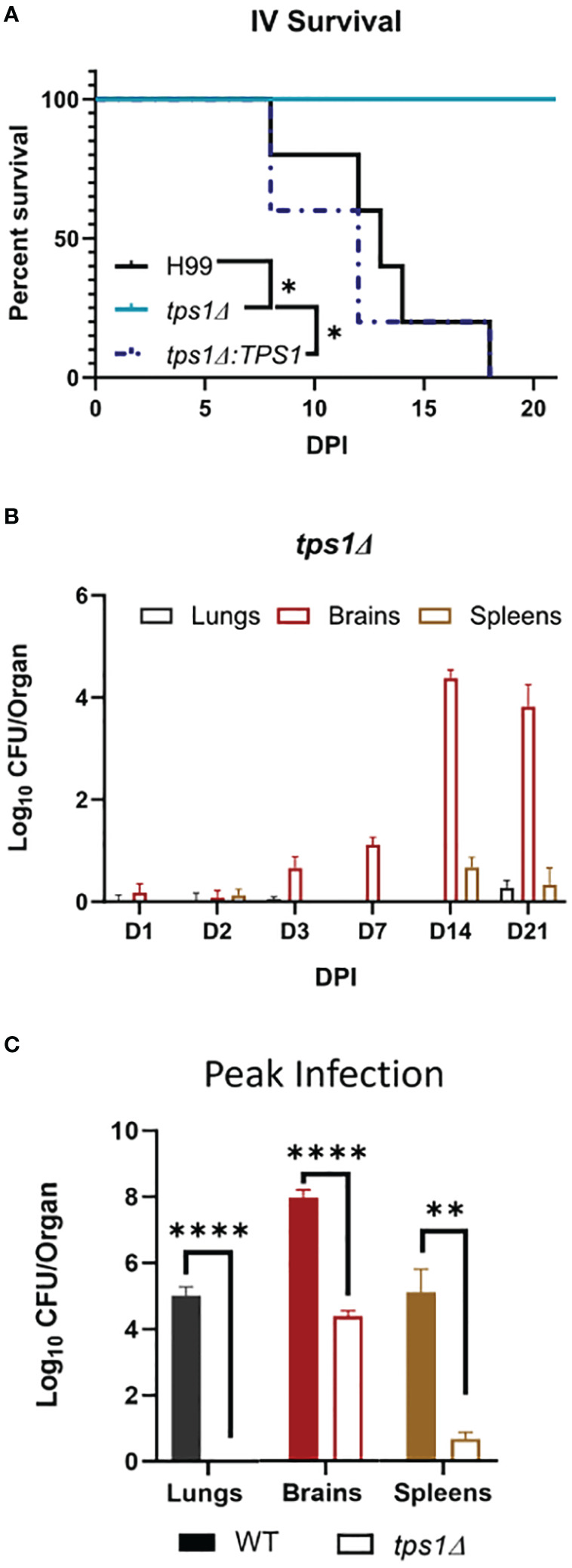
*Cn tps1Δ* remains avirulent in a disseminated infection model with differential level of improved fungal control within specific organs. BALB/c mice were infected intravenously (IV) with 1*10^5^ *Cn* H99 or *tps1Δ* or *tps1Δ:TPS1*. **(A)** Infected mice were monitored for 21 days for survival. All mice infected with *TPS1*-sufficent *Cn* reached endpoint criteria between 8 dpi and 18 dpi, whereas all *tps1Δ*-infected mice survived; n = 3–5 mice/group. **(B)** Fungal burdens were analyzed for *tps1Δ* IV-infected mice at selected time points between 1 dpi and 21 dpi in lungs, brains, and spleens; n = 4–10. Note that *tps1Δ* was largely unable to establish lung or spleen infection; it was delayed in crossing into the CNS but continued to expand in the brain until 14 dpi. **(C)** Fungal burdens in the lungs, brains, and spleens of IV H99-IV-infected mouse were reported at discrete time points between 8 dpi and 18 dpi and cumulatively at the time of death (median 13 dpi). These were compared with the *tps1Δ* IV-infected mice at 14 dpi as that timepoint had the highest CFUs, and these two times are collectively referred to as the peak of infection. n = 3–8 mice/group. *p ≤ 0.05, **p ≤ 0.01, ****p ≤ 0.0001.

**Figure 3:**
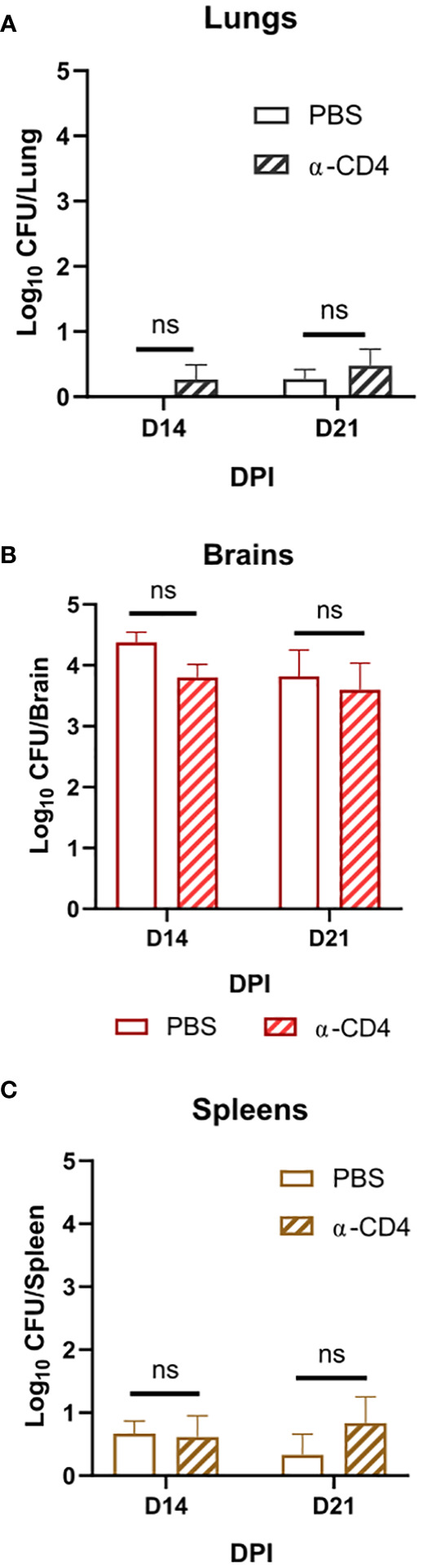
Disseminated *Cn* infection with *tps1Δ* despite organ-specific differences in fungal abundance is controlled in a T-cell-independent fashion in all studied organs. BALB/c mice infected as per **Figure 2**, received injections with anti-CD4 depleting antibody or control (PBS). **(A)** Lung, **(B)** brain, and **(C)** spleen fungal burdens on 14 dpi and 21 dpi. Data shown are the cumulative results of two independent experiments n = 3–10 mice/group. NS, no significance.

**Figure 4:**
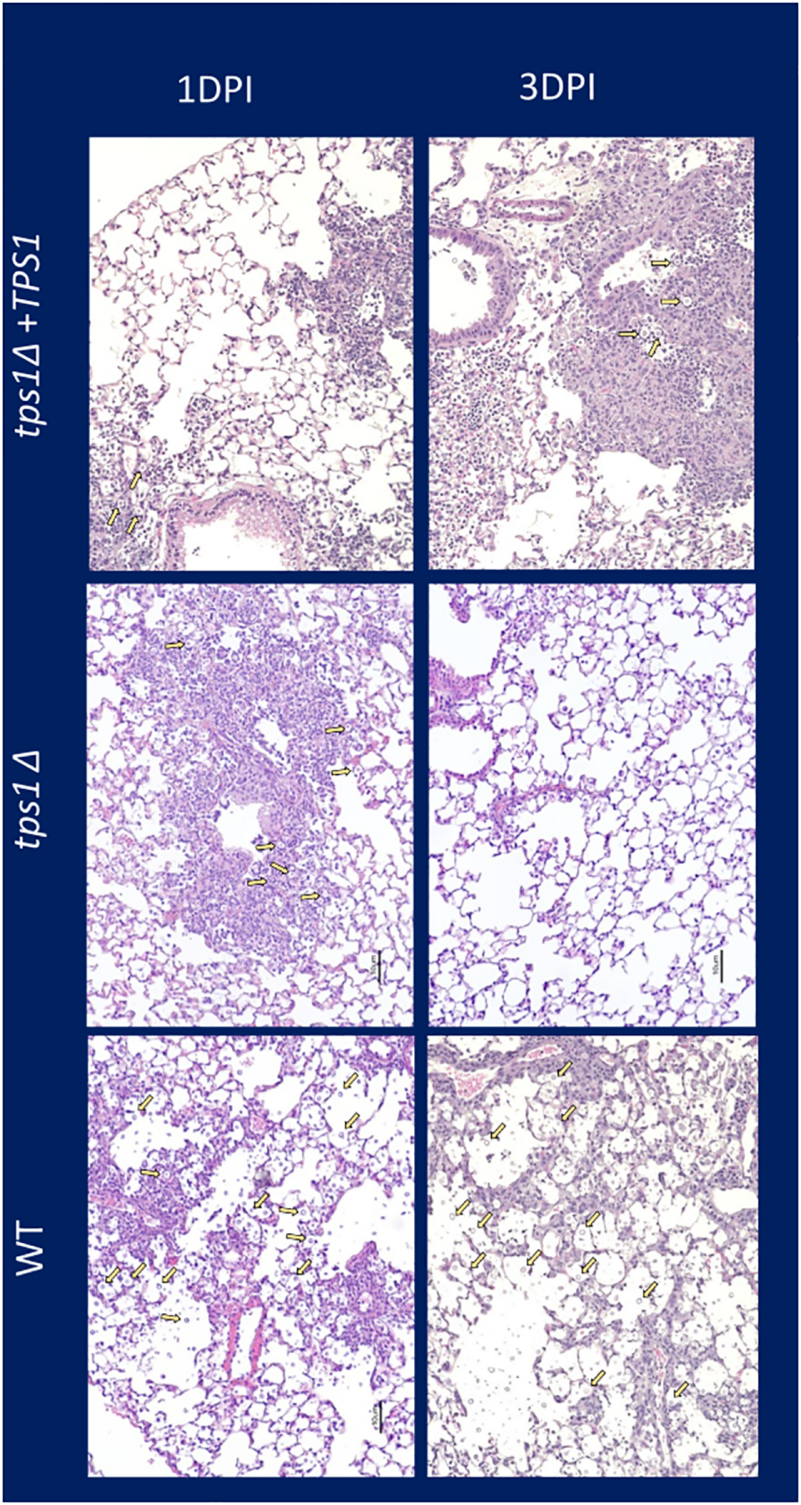
*Cn tps1Δ* generates an initial rapid inflammation in the lungs that quickly resolves the infection. BALB/c mice were infected IT with 1*10^7^ cells of *Cn* H99, *tps1Δ*, or *tps1Δ:TPS1.* H&E-stained histological images of mouse lung at 1 dpi and 3 dpi (10× objective). Note that mice infected with either strain induced similar levels of lung inflammation, but by 3 dpi, *tps1Δ*-infected lungs have mostly contained the infection with minimal inflammation or damage remaining; in contrast, the H99 and *tps1Δ:TPS1*-infected lungs show progressive infection with increasing presence of cryptococcal cells (indicated with yellow arrows), and severe pathology.

**Figure 5:**
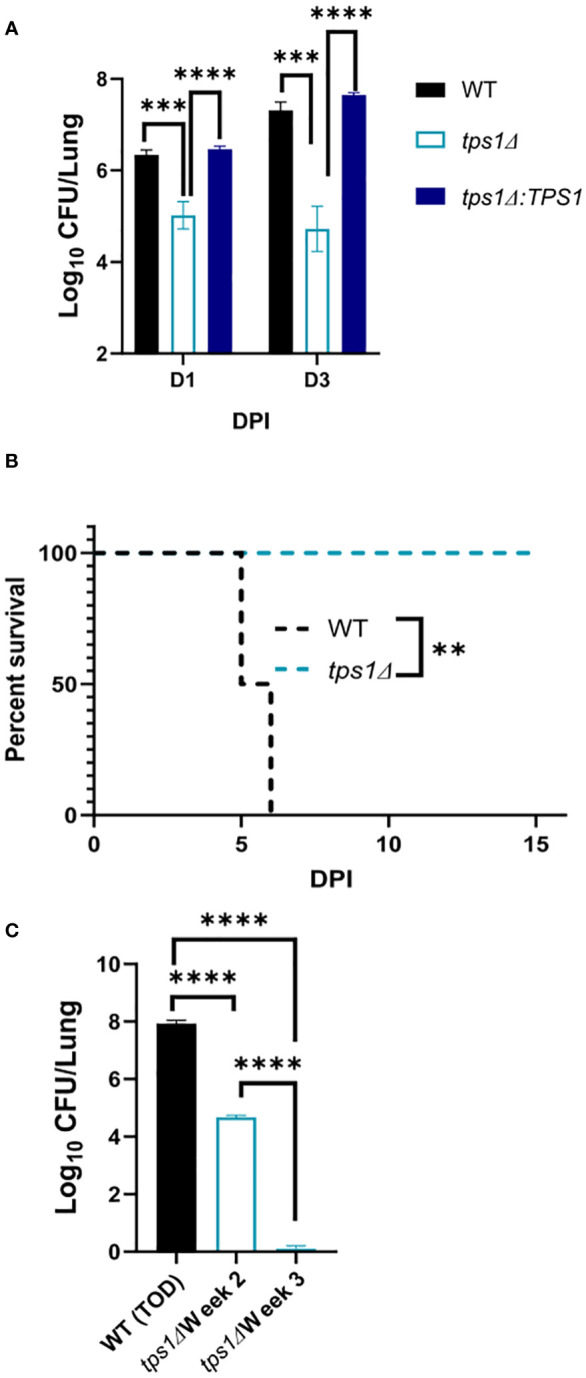
Cryptococcal tps1Δ-gene deletion interferes with *Cn* capsule generation but not urease production. **(A)** 1*10^7^ H99, *tps1Δ*, or *tps1Δ:TPS1-Cn* were plated on Christensen’s urea agar for visual determination of urease production (pink pigmentation). The image is representative of three multiple plates showing no effect of TPS1. **(B)** Images of India ink stains of H99, *tps1Δ*, or *tps1Δ:TPS1* and the acapsular *Cn cap60Δ* (negative control) cultures conditioned for the *in vitro* capsule stimulation (37°C and 5% CO_2_) and recovered from the lungs of 1*10^7^ IT infected BALB/c at 1 and 3 DPI. **(C)** Quantification of the capsule size from the India ink stains. Data shown (individual cell measurements, means ± SEM) of n ≥150 cells per condition. **p ≤ 0.01, ***p ≤ 0.001, ****p ≤ 0.0001.

*C. neoformans* strain H99 (ATCC 208821, WT), *tps1*-gene deleted mutant strain H99 (*tps1Δ*), and reconstituted mutant strain *tps1Δ*, with TPS1 gene restored (*tps1Δ:TPS1*) (Petzold et al., 2006), were grown for 3 days in Sabouraud dextrose broth (Difco) at 30°C and then moved to 37°C overnight after recovery from 10% glycerol frozen stocks stored at −80°C. Yeast cells were washed in PBS, counted on a hemocytometer with trypan blue, and adjusted to a concentration of 1*10^7^ cell/0.03 mL (high, **Figures 4**, **5** and ex vivo data for **Figure 7**) or 1*10^5^ cells/0.03 mL (normal/low, **Figure 1**) prior to the infection. Serial dilutions of the inocula were plated on Sabouraud dextrose agar (SDA) plates to confirm the CFU number of each inoculum. For the intratracheal infections, mice were anesthetized *via* intraperitoneal injections with ketamine/xylazine (100/6.8 mg/kg body weight) prior to infection (Osterholzer et al., 2009b; Stempinski et al., 2022). For the disseminated mode (**Figures 2**, **3**), yeast cells were washed and counted as above and adjusted to a concentration of 1 × 10^5^ cells/0.2 mL just prior to infection. Male and female mice were infected *via* retroorbital intravenous injection under inhaled isoflurane anesthesia (Neal et al., 2017; Goughenour et al., 2021).

**Figure 6:**
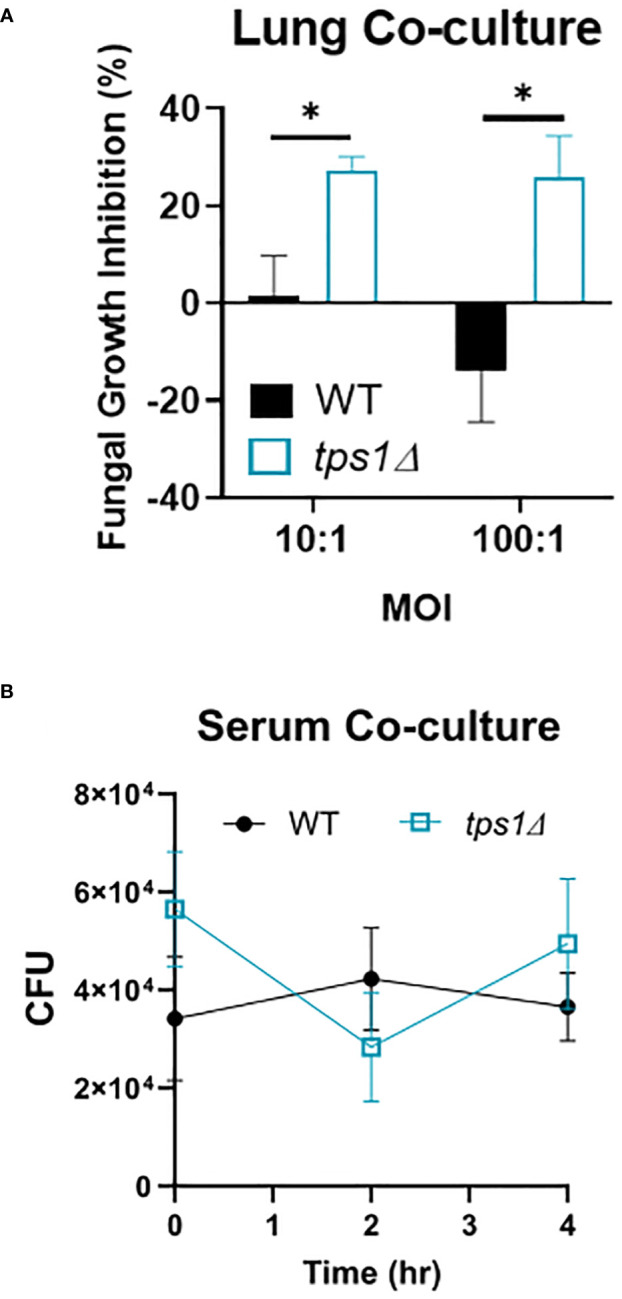
The *Cn tps1Δ* remains avirulent in the lungs, even at the extremely high inoculum. BALB/c mice were infected IT with 1*10^7^ cells of *Cn* H99, *tps1Δ*, or *tps1Δ:TPS1.* Fungal lung burdens **(A)** at corresponding timepoints (three experiments; n = 7–11 mice/group) and the survival analysis **(B)** (n = 4–5 mice/group) support the histological findings. **(C)** Lung fungal burdens evaluated for H99 at the time of death (5 dpi–6 dpi) show its progressive expansion. Lung fungal burdens of *tps1Δ*-infected lungs were evaluated at 2 weeks postinfection, showing fungal containment and 3 weeks postinfection, showing fungal clearance from the lung; n = 3–4 mice/group. *p ≤ 0.05.

### Fungal burdens

Organs were dissected using sterile instruments and homogenized in 2 mL of sterile distilled water. Fungal burden for each organ (lungs, brains, and spleens as indicated in the figures) was quantified by seven-step 10-fold serial dilution of organ homogenate in sterile distilled water, followed by plating of 10 μL of homogenate at each dilution level (undiluted—1:10^7^ dilution) on SDA. CFU per organ was determined by averaging two replicate platings. When required to determine clearance, all 2 mL of organ homogenate was plated on SDA.

### CD4 depletion

Per our labs’ established T-cell depletion method (Neal et al., 2017), mice were dosed with either an anti-CD4 monoclonal antibody (Bio X Cell) on day −3 (pre-infection), day 0 (day of infection), and days 7, 14, 21, 28, and 35 postinfection. Animals in the treatment group received 300 µg of anti-CD4 antibody (clone GK1.5) in 200 μL of sterile PBS, whereas those in the control group received 200 mL of sterile PBS. This depletion protocol was performed in both the intratracheal (**Figure 1C**) and disseminated (**Figure 3**) infection context.

### Histology

Lungs IT infected with H99, *tps1Δ*, or *tps1Δ:TPS1* at 1*10^7^ cells (high) were removed and fixed inflated with 10% neutral buffered formalin on 1 and 3 days postinfection. They were then paraffin embedded and cut into 5-mm sections for H&E staining. The sections were visualized by light microscopy with microphotographs taken by the Digital Microphotography system DFX1200 with ACT-1 software (Nikon, Tokyo, Japan).

### Dispersed lung coculture

Lungs from uninfected mice we digested as per our established protocol (Osterholzer et al., 2009b). In brief, the lungs were removed, minced with scissors, and digested enzymatically at 37°C for 30 min in digestion buffer [RPMI, 5% fetal calf serum, antibiotics, and 1 mg/mL collagenase A (Boehringer Mannheim Biochemical, Chicago, IL), and 30 μg/mL DNase (Sigma-Aldrich, St Louis, MO)]. The digested lung was dispersed by repeated aspiration through the bore of a 10-mL syringe. The cells were then exposed to a red blood cell lysis buffer (0.829% NH_4_Cl, 0.1% KHCO_3_, 0.0372% Na_2_EDTA, and pH 7.4) for 3 min and then quenched by the addition of 10-fold excess of RPMI. Cells were resuspended and filtered through a sterile 100-μm nylon filter. A 20% Percoll (Sigma) gradient enriched leukocytes from cell debris and epithelial cells. The digested lung cells were enumerated on a hemocytometer following dilution in Trypan blue (Sigma). Cells were diluted in culture buffer (RPMI, 5% fetal calf serum, antibiotics) to a concentration of 2 × 10^6^ cells/mL (10:1 MOI) or 2 × 10^7^ cells/mL (100:1 MOI) and plated at 100 µL in 96-well plates.

*C. neoformans* strain H99 (ATCC 208821) and mutant strain H99 *tps1Δ* strains (Petzold et al., 2006) were grown for 2 days in Sabouraud dextrose broth (Difco) at 30°C. Cells were enumerated on a hemocytometer following dilution in Trypan blue (Sigma) to a concentration of 2 × 10^5^ cells/mL. 100 µL was added to the wells containing dispersed lung cells. The wells were cocultured for 4 h at 37°C in 5% CO_2_. Sterile water was used to lyse the lung cells to end the coculture, and then fungal survival for each well was quantified by a seven-step 10-fold serial dilution in sterile distilled water, followed by plating of 10 μL of homogenate at each dilution level (undiluted 1 × 10^−7^dilution) on SDA. CFU per well was determined by averaging two replicate platings.

### Serum coculture

WT (H99), tps1 KO (*tps1Δ*), or complemented (*tps1Δ*:*TPS1*) *C. neoformans* strains were incubated with murine serum for 4 h at 37°C in 5% CO_2_. Fungal cells were enumerated at time 0 h, 2 h, and 4 h by a seven-step 10-fold serial dilution in sterile distilled water, followed by plating of 10 μL of each dilution on SDA.

### Urease assay

H99, *tps1Δ*, and *tps1Δ*:*TPS1 C. neoformans* strains were grown for 2 days in Sabouraud dextrose broth (Difco) at 30°C. Cells were enumerated on a hemocytometer following dilution in Trypan blue (Sigma) to a concentration of 1 × 10^7^ cells/mL. 10-µL spots were plated on Christensen’s urea agar (Sigma 27048-500G-F) for visual determination of urease production (pink color indicator).

### Capsule

#### *In vitro* stimulation

Capsule stimulation was performed as described (Kumar et al., 2014). Briefly, *C. neoformans* cultures were grown in yeast peptone dextrose (YPD) broth at 30°C for 24 h on a shaker. Cells were washed once in PBS, again in DMEM, and then resuspended and diluted to a concentration of 10^6^ cells/mL in Dulbecco’s modified Eagle’s medium (DMEM). 1 mL of this suspension was then incubated in a tissue culture plate at 37°C in 5% CO2 for 24 h. Following incubation, yeast cells were fixed in 10% formalin. For visualization, these yeast cells were washed in PBS and resuspended in PBS to achieve a concentration of 10^8^ cells/mL. Multiple inductions of the same yeast strain were combined to reach the desired concentration.

#### Ex vivo

Mice were infected with 1 × 10^7^ cells/mL H99 or *tps1Δ C. neoformans* intratracheally as described above. At 1 and 3 days postinfection, mice were euthanized by CO_2_ inhalation. Lungs were removed and placed in a 15-mL tube containing. 2 mL collagenase buffer (1 mg/mL collagenase in PBS) was added to each tube, and the lungs were homogenized. 8 mL collagenase buffer was added, and the tubes were vortexed and incubated for 1 h at 37°C. The samples were washed three times, resuspended in 2 mL of 0.05% SDS and then filtered with a 70-µm filter. The yeast cells were fixed in 10% formalin. For visualization, yeast cells were washed in PBS and resuspended in PBS to achieve a concentration of 10^8^ cells/mL.

#### Capsule quantification by India ink exclusion staining

India ink was added to the cell suspension, and 10 μL of this mixture then spotted onto a glass slide for microscopy. The capsule was visualized by light microscopy with microphotographs taken by the Digital Microphotography system DFX1200 with ACT-1 software (Nikon, Tokyo, Japan). Capsule size measurements were taken using ImageJ.

### Statistical analysis

**Figure 7:**
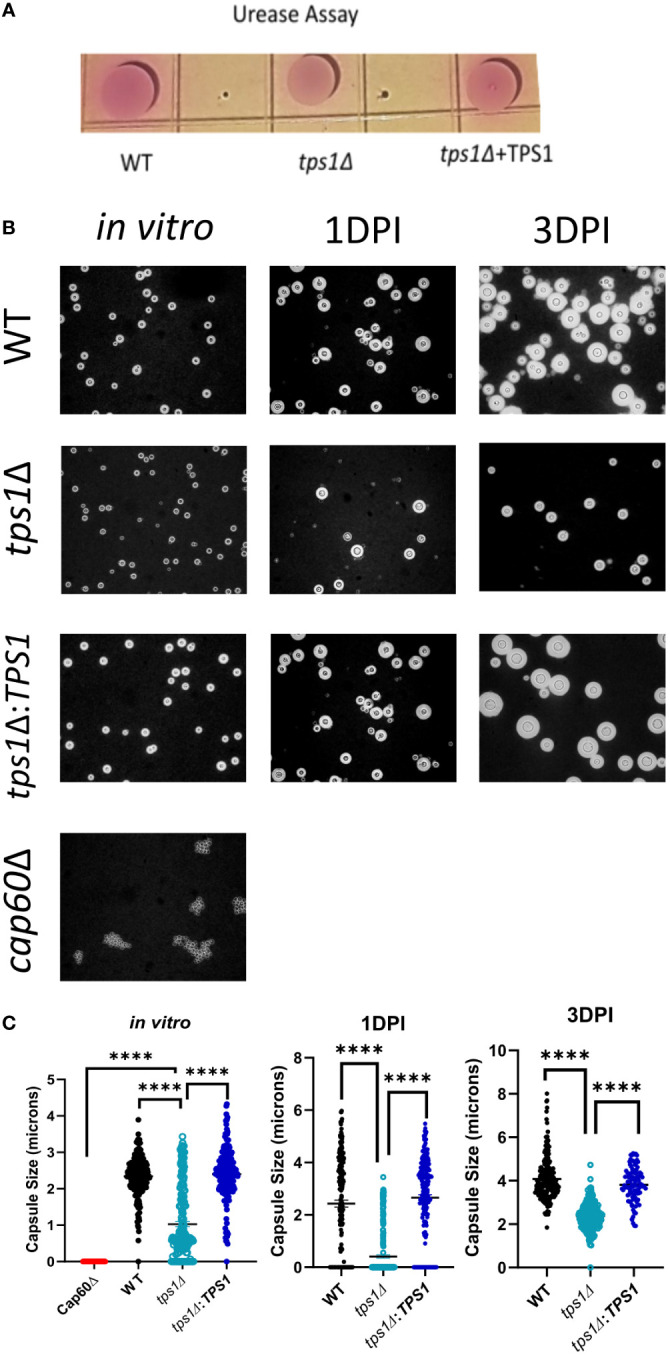
Cryptococcal *tps1*-gene deletion renders *Cn* susceptible to the acute resident pulmonary defenses but not the antimicrobial serum effects. **(A)** Dispersed lung cells enriched in leukocytes were isolated from naïve BALB/c mice and cocultured with either H99- or *tps1Δ Cn* for 4 h at 37°C and plated for surviving fungal numbers. Data shown (means ± SEM) are the cumulative results of two independent experiments (n = 8) and **(B)** H99 or *tps1Δ Cn* was incubated with serum for 0 h–4 h with viability assessed every 2 (h) No differences were seen in survival in any of the strains. ****p ≤ 0.0001.

Values are reported as means with variance indicated. GraphPad Prism v8 software (GraphPad Software, San Diego, CA) was used to perform statistical analyses. Mantel–Cox tests were used for survival experiments (**Figures 1B**, **2A**, **5A**). Unpaired Student t test (two-tailed test) was used for comparisons of individual means (**Figures 1C**, **2C**, **3**, **5A, C**, **6A**); Welch’s t-test (**Figure 1B**) and one-way ANOVA were used for multiple comparisons (**Figure 7C**). Data in all figures are the means ± SEM (unless otherwise indicated). Values that are statistically significant are indicated in the figures as follows: *p ≤ 0.05, **p ≤ 0.01, ***p ≤ 0.001, ****p ≤ 0.0001.

## Results

### *Cryptococcal tps1*-deletion results in *C. neoformans’* failure to develop a persistent infection in the lungs and to resist its innate defenses

The requirement of cryptococcal *tps1* gene expression for virulence of *C. neoformans* was demonstrated in several invertebrate and vertebrate models of infection (Petzold et al., 2006). However, little is known about the role of this pathway for fungal interactions with the host, including its ability to survive long-term in the mammalian host and its contribution to dissemination. To establish these parameters, we evaluated the role of *tps1* in a group of murine models of mouse infection. Our first study tested whether *tps1Δ* would be sufficient to render *C. neoformans* virulent in the highly susceptible C57Bl/6 mice as compared with BALB/c mice, which show improved resistance to pulmonary infection with *C. neoformans* (Zaragoza et al., 2007; Chen et al., 2008). WT (H99) or *tps1* KO (*tps1Δ*) *C. neoformans* (1 × 10^5^ cells/mL) were instilled intratracheally, as described earlier (Chen et al., 2008) to perform parallel inoculations in BALB/c and C57Bl/6 mice, and mouse survival was evaluated (**Figure 1A**). While more sensitive C57BL/6 succumbed to infection with WT *C. neoformans* (reached endpoint criteria between 14 dpi and 21 dpi) consistent with our previous report (Osterholzer et al., 2009b), all *tps1Δ*-infected mice survived until day 28 with no remaining fungus in the lung at this time. In this respect, deletion of cryptococcal *tps1* had a similar effect in C57BL6 mice as in more resistant BALB/c, which survive up to 25 days with the H99 WT strain (Zhang et al., 2009; Eastman et al., 2015) and completely withstand *tps1Δ* (**Figure 1A**).

Considering that *tps1Δ* was equally required for virulence in both strains of mice, we analyzed the effect of cryptococcal *tps1Δ* on fungal clearance rate from the infected lungs. We performed infections with H99, *tps1Δ*, or *tps1Δ:TPS1* (complement) strains in BALB/c mice (**Figure 1B**) and analyzed pulmonary fungal burdens at 1, 3, and 7 days postinfection (dpi). The *tps1Δ C. neoformans* significantly reduced fungal burdens in the lungs as soon as 24 h postinfection, continuing to decrease at day 3, and completely cleared the fungus from the lungs by 7 dpi. In contrast, the H99 and *tps1Δ:TPS1* cryptococcus strain established a persistent infection in the lungs. The rapid clearance of the *tps1Δ* strain suggested that the rapid fungal clearance of *tps1Δ C. neoformans* likely occurred independently of the adaptive immune response. To test this, we performed a CD4-T-cell depletion with anti-CD4 antibody in BALB/c mice, as reported previously (Neal et al., 2017), and then infected with *tps1Δ* or H99 (**Figure 1C**). As expected, the T-cell depletion at this early time point of infection did not result in any changes in the expansion of the WT strain H99 but also did not affect the trajectory of *tps1Δ* lung burden. Thus, cryptococcal *tps1* resulted in *C. neoformans* failure to develop persistent infection in the lungs and enabled the host to rapidly clear the lung infection without the help from CD4+ T cells.

### *Cryptococcal tps1-*gene deletion renders the fungus avirulent in a disseminated model of infection; however, there is differential site-specific control.

Due to the rapid and complete elimination of the *tps1Δ C. neoformans* from the infected lungs in a pulmonary infection model, we could not evaluate the role of *tps1Δ* in other organs to which the fully virulent fungus disseminates. As such, we used our disseminated infection model achieved *via* an intravenous infection (Neal et al., 2017) that bypasses the pulmonary defenses. The WT, *tps1Δ*, or *TPS1*-complemented *C. neoformans* 10^5^ cells were injected IV into BALB/c mice, and mouse survival was evaluated (**Figure 2A**). All WT and complemented *C. neoformans*-infected mice reached endpoint criteria between 8 and 18 DPI, whereas all *tps1Δ*-infected mice survived for an observed 21 days. To determine the specific requirement of *tps1Δ* for *C. neoformans* to establish infection at specific target organs (**Figure 2B**), we quantified fungal burdens in the lungs (gray), brains (red), and spleens (tan) at 1 dpi, 2 dpi, 3 dpi, 7 dpi, 14 dpi, and 21dpi infected with *tps1Δ C. neoformans*. The *tps1Δ C. neoformans* strain is unable to reach more than a transient low level (<100 cells) of infection in the lungs and spleens. WT H99 *C. neoformans* establishes a progressive infection in these tissues (fungal burdens reported in **Supplementary Figure 1**) reaching ~10^5^ CFU/lungs and spleens by the time of death. Even at the peak of infection (time of death for WT and 14 dpi for *tps1Δ*), the *tps1Δ* strain still shows severe attenuation compared with the WT (**Figure 2C**). Interestingly, despite this severe attenuation, the *tps1Δ C. neoformans* is capable of entering the CNS compartment and establishing a low-level infection in the brain rather than be cleared (**Figure 2B**). The *tps1Δ* strain increased from low-level brain infection, from 10^1^ CFU at 7 dpi to around 10^4^ CFU/brain at 14 dpi to a slight taper off on 21 dpi, a timing consistent with the development of the adaptive response in the brain in this model (Neal et al., 2017). However, our follow-up experiment excluded the role of CD4+ T cells, in this delayed control of *tps1Δ* strain. Mice treated with either anti-CD4 depleting antibody or received control IP injections (PBS) showed comparable fungal burdens on day 14 and day 21 postinfection in the lungs (**Figure 3A**), brains (**Figure 3B**), and spleens (**Figure 3C**). Thus, cryptococcal *tps1Δ* results in an improved control of the fungus in multiple organs, with somewhat less effective control in the brain, but in all cases independent of T-cell-mediated responses.

### *C. neoformans tps1Δ* fails to overwhelm the pulmonary defenses, even at the extremely high inoculum

The very rapid suppression of the *tps1Δ C. neoformans* in the lungs infected with lower inoculum, typically used in most studies, would make the direct comparison of lung pathology very challenging as the *tps1Δ* strain would not rise to the sufficiently large fungal burdens to be analyzed by histology. Thus, we increased the inoculums to 10^7^ cells and performed follow-up IT-infection studies. Histological analysis of BALB/c lungs infected with the WT, *tps1Δ*, or complemented *C. neoformans*-infected mice revealed that all three strains were present in the alveolar space and induced a robust inflammatory response by 1DPI (**Figure 4**). In the lungs infected with any of the three strains we observe abundant leukocytes that migrated to the alveolar airspace, especially in peribranchial areas of the lungs. However, by 3 DPI, the mice infected with the *tps1Δ* strain show few visible *C. neoformans* cells compared with the *tps1*-sufficient strains in the airspace and much lower level of inflammation. The WT and complemented-strain-infected lungs show a progressing infection and an ongoing inflammatory response, characteristic for cryptococcal pneumonia (Osterholzer et al., 2009b). Consistent with the histology data, we found a roughly one-log difference in fungal burden at day 1 between the *tps1Δ* strain and *tps1*-sufficient strains. This progressed to a 2-log difference in CFU on day 3 postinfection between the *tps1*-sufficient and *tpsΔ* strains in the infected lungs (**Figure 5A**). The follow-up survival studies showed rapid mortality of the WT-strain-infected mice, which all succumbed within 6 DPI, whereas the *tps1Δ*-infected mice showed no apparent symptoms during 15 DPI of observation and a 100% survival (**Figure 5B**). Our lung CFU analysis at the conclusion of the survival study showed 10^8^ CFU at the time of death in mice infected with the WT and a substantial clearance of *tps1Δ* from the infected lungs down to 10^4^ CFU/lung on day 14 (**Figure 5C**). This clearance kinetics was slower compared with the low-dose *tps1Δ*-infected mice, which cleared the infection by day 7, but ultimately, we saw the complete clearance of the mutant from the lungs by day 21 (**Figure 5C**). Together, these data show that while the resident lung defenses could be temporarily overwhelmed by the extremely high inoculum of *tps1Δ C. neoformans*, this strain remained avirulent, unable to cause severe lung pathology, and was ultimately cleared by the infected lungs.

### *Cryptococcal tps1*-gene deletion renders the yeast susceptible to the resident lung defenses, but not to humoral components of serum

The extremely rapid and effective suppression of the *tps1Δ C. neoformans* in the lungs but not in the brains led us to test whether there were lung-specific/resident defenses that restrained the trehalose-deficient strain. We analyzed the short-term effect on *C. neoformans* survival in a coculture with dispersed lung cellular components, which would exclude the effects of recruited immune components. CFUs of WT and *tps1Δ* cryptococci were evaluated at 4 h post-incubation compared with the fungi incubated in media alone to calculate percent inhibition (**Figure 6A**). Even after this short-term incubation, the *tps1Δ* yeasts were suppressed by lung cells (20%–25% inhibition) whereas WT cells were uninhibited. To account for possible effects of humoral defenses (such as complement or antibodies) we performed similar incubation of the WT or *tps1Δ* cryptococci in mouse serum (**Figure 6B**). No differences in fungal survival were detected during the 4 h of incubation, indicating that *tps1Δ* strain did not render the fungus susceptible to the humoral serum components present in the naive mouse serum. Thus, trehalose biosynthesis provides protection against resident lung defenses present in the lungs at the time of infection, but not against the serum humoral components.

### Trehalose biosynthesis is required for optimal capsule formation by *C. neoformans*

While TPS1 enzyme is unlikely to directly interfere with host defenses, it has been proposed to be an upstream element required for synthesis of other known virulence factors, which in turn affect the host defenses. Some effects of *tps1Δ* for several cryptococcal virulence factors activities were studied (Petzold et al., 2006; Ngamskulrungroj et al., 2009), but the strong early lung-specific survival defect in *tps1Δ* prompted us to reevaluate factors that allow the fungus to be better adapted to pulmonary defenses. Urease production by *Cryptococcus* is one of fungal pulmonary virulence factors we studied previously (Osterholzer et al., 2009b), and thus we evaluated the urease production in WT, *tps1Δ*, or complemented *Cryptococcus* strains on Christensen’s urea agar (**Figure 7A**). All three *C. neoformans* strains, including *tps1Δ*, showed the robust and equivalent production of urease indicated by pink color conversion of the colonies (**Figure 7A**). We next explored the effect of tps1Δ on cryptococcal capsule formation, as this virulence factor is important for the yeast to efficiently withstand the innate host defenses (Casadevall et al., 2019). While *tps1Δ C. neoformans* strain was reported to produce capsule *in vitro* (Petzold et al., 2006; Ngamskulrungroj et al., 2009), the effect of *tps1* on capsule size in culture and capsule formation in the lung environment *in vivo* has not been investigated. We cultured WT, *tps1Δ*, and complemented strains *in vitro* using capsule stimulating conditions, in addition to recovering fungal cells from the lungs from the infected animals at 1 DPI and 3 DPI, we performed India ink stains to visualize the capsule (**Figure 7B**). All three strains produced visible capsule compared with the acapsular *cap60Δ* control (Chang and Kwon-Chung, 1998). However, the capsule produced by the *tps1Δ* strain was visibly less robust than that produced by WT cells and this difference became particularly apparent in the lungs at 3 DPI. The capsule size measurements *in vitro* and *ex vivo* showed highly significant reduction of the capsule size in *tps1Δ* strain (**Figure 7C**). The capsule of the *tps1Δ Cryptococcus* was at least half the size of that of the WT. Thus, whereas *tps1Δ Cryptococcus* strain can generate a capsule both in capsule stimulating conditions *in vitro* and expands its size over time in the murine lungs, the capsule generated is not as robust, providing a potential explanation for the increased susceptibility of *tps1Δ* strain to the resident and innate mechanisms of host defenses, especially in the lungs.

## Discussion

The requirement of trehalose biosynthesis for fungal virulence is an area of growing research (Petzold et al., 2006; Martínez-Esparza et al., 2007; Al-Bader et al., 2010; Magalhães et al., 2017; Washington et al., 2023). Previous studies have established the requirement for trehalose biosynthesis in *C. neoformans* virulence in several invertebrate and vertebrate models of infection (Petzold et al., 2006) but have not defined how trehalose impacts host–pathogen interactions for this pathogen and its ability to persist long-term within the host. Here, we provide novel insights into how interception of the trehalose pathway affects these interactions by demonstrating that cryptococcal TPS1 expression 1) enables the host to control an otherwise progressive and lethal *C. neoformans* infection in pulmonary and disseminated stages of infection; 2) abolishes the requirement for CD4 T cells for *C. neoformans* control in both pulmonary and disseminated/CNS infection models; 3) renders the fungus highly vulnerable to the local/resident mechanisms of pulmonary host defenses; and 4) interferes with cryptococcal capsule formation, providing a potential mechanistic underpinning for the modified interactions listed above.

Our data demonstrate the requirement for *tps1* for *C. neoformans* to avoid host fungal control and cause progressive and lethal infection in pulmonary and disseminated stages of infection. We build upon previous work showing *tps1Δ* in *C. neoformans* results in loss of virulence in murine, immunosuppressed rabbit, and *Caenorhabditis elegans* models (Petzold et al., 2006). We are now showing that TPS1 is required for virulence in two murine models of pulmonary infection and a murine disseminated model (**Figures 1A**, **2A**). We found that the *tps1* deletion rendered *C. neoformans* avirulent even in the C57BL/6 mouse strain, known to be more susceptible to cryptococcosis as compared with the more resistant BALB/c mice (Zaragoza et al., 2007; Chen et al., 2008). We found that this requirement for *tps1* gene in *C. neoformans* infection was particularly important in the pulmonary compartment. The *tps1Δ* mutant was rapidly cleared by the innate immune system in the lungs during pulmonary infection (**Figure 1B**). While a low level of fungi was able to survive in the lungs for the first 3 days, all the *tps1Δ C. neoformans* colonies were cleared by 7dpi when standard infection inoculum was used. Even when we overwhelmingly challenged the lungs with an extremely high fungal load (triggering mouse death in 5 dpi–6 dpi with H99 groups), *tps1Δ* was cleared over time by the host defenses (**Figure 5C**). This tight control of *tps1Δ* growth by the lungs was likewise apparent in our disseminated cryptococcosis model, where the strain was never able to establish an infection in the lungs bypassing the lungs in contrast with H99 (**Figure 2B**). In addition, we show that *tps1* is further required for the virulence composite even when this pulmonary control is bypassed in the disseminated model. The *tps1Δ* strain was severely attenuated in the brain where it had fungal burdens four to five logs lower than the WT (**Figure 2C**). However, the *tps1Δ C. neoformans* was able to cross from the blood to the central nervous system (CNS) and establish a low-level subclinical infection in the brain. These fungal burdens never reached the three orders of magnitude and lethal fungal burden of the WT at week 2 and week 3, beyond the timepoint of mortality of the H99-infected mice. We therefore conclude that the brain immune response can still control *tps1Δ* strain. Interestingly, our disseminated model findings contrast previous findings in an immuno-compromised rabbit model, which showed a complete loss of *tps1Δ Cryptococcus* CFUs in cerebrospinal fluid (CSF) (Ngamskulrungroj et al., 2009). This could be attributed to the higher temperature sensitivity of *tps1Δ C. neoformans* and the immune system differences between these two models. For instance, rabbits have a higher body temperature, in the range of 38.6°C–40.1°C, compared with 36.2°C–37.5°C in mice (Hankenson et al., 2018; Table Normal Rectal Temperature Ranges, n.d.) and the notably different subsets of the immune cells. These two components likely account for even more dynamic control of the *tps1Δ* strain in the rabbit meningitis model.

Our identification of the rapid clearance of the *tps1Δ* strain provided a clue that host control of *tps1Δ C. neoformans* may occur independently of the adaptive immune response. To test this hypothesis, we performed a CD4-T-cell depletions with anti-CD4 antibody in BALB/c mice, as reported previously (Neal et al., 2017) and followed with *tps1Δ* mutant or H99 infection (**Figure 1C**). As expected, the CD4 T-cell depletion at this early time point of infection did not result in any changes in the expansion of strain H99 but also had no effect on the already suppressed *tps1Δ* lung burden. Neither have we found any contribution of CD4+ T cells to fungal control at later time-points in our disseminated model, where lungs were able to prevent *tps1Δ* fungal invasion in the T-cell-depleted mice at 2 and 3 weeks postinfection (**Figure 3B**). It is well established that *Cryptococcus* in the brain requires the adaptive, CD4+ T-cell-mediated immunity for anti-cryptococcal defenses (Uicker et al., 2006; Neal et al., 2017, Guo et al., 2022). Still brain invasion of *tps1Δ C. neoformans* in our disseminated model was significantly delayed (2- and 3-week postinfection time points). We excluded the possibility that T cells were required to contain *tps1Δ* strain in the brain but not the lungs, finding no difference in its brain containment following global CD4^+^-cell depletion (**Figure 3A**), supporting the notion that the *tps1Δ* strain was completely independent of CD4+ T-cell presence, regardless of target organ or infection model. Thus, cryptococcal *tps1Δ* the host to clear the lung infection bypassing conventional adaptive immune mechanisms.

Our data support that host’s control of the trehalose-deficient mutant *Cryptococcus* is driven by the elements of the resident (**Figure 6A**) and quite likely recruited innate immune system in the lungs (**Figure 4**). We have established that pulmonary control of *tps1Δ C. neoformans* strain is rapid (**Figure 1B**) and independent of the CD4+ T-cell-driven adaptive immune response (**Figures 1C**, **3**). We show that mice began to control trehalose-deficient *C. neoformans* right from the start showing a substantial suppression already within the first day postinfection implicating a strong role for resident mechanisms of fungal control (**Figure 1B**). We further see that the *tps1Δ* strain is inhibited by coculture with dispersed lung cellular components, which are limited to lung-specific/resident defenses and contain no bloodstream recruited components (**Figure 6A**). On the other hand, our histological results show that *tps1Δ C. neoformans*-infected mice induced a robust inflammatory response by 1 DPI that largely resolves by 3 DPI (**Figure 4**), corresponding with the initial wave of fungal clearance, which removes over 99% of the instilled fungus and its total removal by day 7 (low dose). This timing overlaps with the previously defined “innate phase” of the immune response in the murine lungs (Huffnagle et al., 1996; Osterholzer et al., 2009a; Eastman et al., 2015). One explanation of these findings is that *tps1Δ C. neoformans* stimulates an increased immune response by the recruited innate immune system that results in increased recruitment of all or specific subsets of innate immune cells. This could explain the increased control of the mutant strain. However, further studies are required to characterize any differences in the innate immune response to *tps1Δ C. neoformans.*

Since the lung is a main host barrier that *Cryptococcus* needs to breach (Curtis et al., 1994; Huffnagle and Lipscomb, 1998; Osterholzer et al., 2009a; Pappas, 2013), the ability of the fungus to survive in this niche is critical to pathogenesis. While the pulmonary factors to which TPS1-deletion stain is susceptible remain unknown currently, studies are underway to investigate specific pulmonary factors that efficiently drive control of the *tps1Δ* strain. When the pulmonary control was bypassed in our disseminated model, we found that the lungs and spleens could control *tps1Δ Cryptococcus*, but the fungus managed to enter the brains, although to a much lesser extent than the WT fungus. This outcome signifies that TPS1 is not necessary for fungal crossing the blood–brain barrier (BBB) and to survive within the CNS at last for some period of time. We are left with the question of why the lungs are able to quickly control *tps1Δ Cryptococcus* through resident and early innate mechanisms, whereas the brain cannot. A recent study indicated that the major resident host immune cell in the brain, the microglia, are not effective at controlling and provide a niche for growth of *C. neoformans* H99 in the brain early in the infection (Mohamed et al., 2023). One possible explanation is that microglia alone are not sufficient to kill *tps1Δ C. neoformans* in the brain and it takes a much longer time for the host to recruit the proper cellular defenses against the fungus in this tightly controlled immune privileged environment and future studies are needed to address this point.

Finally, our data show a novel link between trehalose biosynthesis and capsule production in *C. neoformans.* The formation of the cryptococcal capsule is perhaps the most well-established virulence factor for this fungus (Fromtling et al., 1982; Chang and Kwon-Chung, 1994). When capsule formation in trehalose-deficient cryptococcus was first investigated, it was observed that *tps1Δ C. neoformans* strain was capable of capsule production in the *in vitro* conditions (Ngamskulrungroj et al., 2009). However, when *tps1* gene was deleted in the closely related species *Cryptococcus gattii*, the fungus became acapsular (Ngamskulrungroj et al., 2009). As expected from an acapsular mutant, the *tps1Δ C. gattii* showed full pulmonary clearance within 1 week of infection (Ngamskulrungroj et al., 2009). This clearance rate parallels that of *tps1Δ C. neoformans*, in our study, which prompted us to look whether the observed severe defects in pulmonary virulence of *tps1Δ* could be linked to the capsule. We found that the capsule size *in vitro* and in the lungs of the *tps1Δ*-infected mice was at least half the size of that of the *tps1*-sufficent WT and complemented strains (**Figure 7C**). Interestingly, trehalose is not a known structural component of the cryptococcal capsule. Therefore, future studies are needed to uncover how the TPS pathway contributes to the capsule formation. One possible hypothesis is that *tps1*-deleted mutants without Trehalose-6-phosphate production as a signaling molecule have cell wall alterations, which in turn affect the capsule formation/attachment. In fact, altered cell wall was reported in *Candida albicans* tps1/tps1 null mutant (Thammahong et al., 2017) and *Aspergillus fumigatus* (Al-Bader et al., 2010). Mutants with an altered cell wall structure show significant stress sensitivity and virulence defects just like the acapsular strains (Reese and Doering, 2003; Reese et al., 2007; Rodrigues et al., 2018), and more research is needed to determine how the *TPS* pathway and formation of trehalose-6-phosphate contributes to capsule formation in *C. neoformans*.

In conclusion, this study has found that TPS1 in *C. neoformans* is required to provide fungal protection against host defenses in the lungs. Host control is driven by resident/innate factors not humoral, or T-cell-mediated responses. TPS1 provides protection against these host defenses likely in part through the generation of a fully robust capsule. The deletion of *tps1* creates an opportunity for more effective immune attack executed by the innate host defense elements. Thus, inhibition of the TPS1 enzyme could serve as a way to incapacitate the fungi as a therapeutic approach even in patients lacking adaptive immune responses.

## Data availability statement

The original contributions presented in the study are included in the article/**Supplementary Material**. Further inquiries can be directed to the corresponding authors.

## Ethics statement

The animal study was approved by Veterans Affairs Institutional Animal Care and Use Committee. The study was conducted in accordance with the local legislation and institutional requirements.

## Author contributions

KG: Formal analysis, Investigation, Methodology, Writing – original draft, Writing – review & editing. AC: Investigation, Writing – review & editing. JX: Conceptualization, Writing – review & editing. XH: Investigation, Writing – review & editing. RH: Investigation, Writing – review & editing. CG: Conceptualization, Writing – review & editing. JT: Conceptualization, Writing – review & editing. DT: Conceptualization, Writing – review & editing. JP: Conceptualization, Writing – review & editing. MO: Conceptualization, Funding acquisition, Methodology, Writing – review & editing.
