# Supplementary material for: Cryptococcus neoformans trehalose-6-phosphate synthase (tps1) promotes organ-specific virulence and fungal protection against multiple lines of host defenses
Source: Front Cell Infect Microbiol. 2024 May 22;14:1392015. doi: 10.3389/fcimb.2024.1392015 (PMC11150607; doi:10.3389/fcimb.2024.1392015)
Supplement: Supplementary file 1 [file DataSheet_1.docx]

**Supplement Fig. 1**

**Supplement figure 1. Cn H99 rapidly and progressively establishes infection in multiple organs following IV injection.**

BALB/c mice were infected intravenously (IV) with 1*10^5 Cn H99. Fungal burdens were analyzed for H99 IV-infected mice at selected time points between 1-7dpi and the time of death (TOD; median 13dpi) in lungs (gray), brains (red), and spleens (gray); n=4-10. H99 rapidly establishes infection in all 3 organs although highest burdens are seen in the brain.
